# Functional assessment and outcome following surgical treatment of displaced tibial plateau fractures: a retrospective analysis

**DOI:** 10.1007/s00068-023-02401-x

**Published:** 2023-11-17

**Authors:** Patrick Gahr, Thomas Mittlmeier, Anja Grau, Philipp K. E. Herlyn, Anja Rahn, Dagmar-C. Fischer

**Affiliations:** 1 Department of Traumatology, Hand- and Reconstructive Surgery, University Medical Center, Rostock, Germany; 2 Department of Pediatrics, University Medical Center, Rostock, Germany; 3 Present Address: Department of Internal Medicine II, Klinikum Südstadt Rostock, Rostock, Germany; 4 Present Address: Department of Traumatology, Hand- and Reconstructive Surgery, Städtisches Klinikum Dresden-Friedrichstadt, Dresden, Germany

**Keywords:** Tibial plateau fracture, Fracture classification, Functional outcome, KOOS, Patient-reported outcome measures, Dynamic plantar pressure analysis

## Abstract

**Purpose:**

Patients with tibial plateau fractures (TPF) are at risk of long-term hampered bipedal locomotion. A retrospective single-center study using patient-related outcome measures and a sophisticated assessment of walking abilities was conducted.

**Methods:**

Adults receiving surgical treatment of an isolated TPF between January 2012 and December 2016 received the KOOS questionnaire together with the invitation for an extensive follow-up examination on the clinical outcome including standardized assessment of the walking abilities (loadsol^®^ system). Outcome was assessed relative to the severity of the injury or time to follow-up. Fractures were classified according to AO/OTA and Luo, respectively.

**Results:**

58 out of 132 eligible patients filled in the questionnaire and participated at a median follow-up of 3.05 years after injury. For the categories “pain”, “mobility”, and “daily life activities”, all patients were rather satisfied and this was virtually not related to the time between fracture and assessment. Relevant limitations were reported for “sports and recreational activities” and “quality of life”. Loading of the previously fractured leg was most evidently changed on stairs and outdoor walking. Outcome was not related to either fracture type severity or time from injury.

**Conclusion:**

Outcome after an isolated TPF is neither related to fracture type, severity of the fracture nor time from injury. Simple gait analysis techniques relying on different tasks appear to yield a more sophisticated image on functional deficits after TPF than classical exam of ground-level walking and correlate quite well with validated patient-related outcome measures as the KOOS.

**Supplementary Information:**

The online version contains supplementary material available at 10.1007/s00068-023-02401-x.

## Introduction

Tibial plateau fractures (TPF) account for roughly 1% of all fractures and require medial, lateral, and/or axial loading via the femoral condyle [1–4]. Usually, the fracture patterns are complex and the therapy can be challenging, i.e., to reconstruct the joint surface, the knee axis, and a “height stable” tibial plateau without prolonged immobilization of the joint. All of these factors need to be considered to prevent long-term complications and impairment of the knee, i.e., posttraumatic accelerated degenerative joint disease [2, 5, 6]. Thus, the planning of the treatment is based on both a two-dimensional radiography assessment and an computed tomography to allow a detailed analysis of the fracture pattern and selection of the preferred surgical approaches. Beyond this, these imaging modalities are used for classification of the fracture, i.e., to allow comparison of treatment and outcome across surgeons and in view of the severity of the fracture. Recently, we provided data to a large retrospective multicenter analysis directed to investigate the putative impact of the fracture classification according to the AO/OTA or the three columns concept [7] on the choice of the surgical approach [8]. At the time we retrieved the data for this analysis from our data management system, patients were invited to score the outcome and to participate in an examination of the current mobility and their walking abilities under conditions of daily life. Furthermore, this approach allowed us to seek for any correlation between the morphological characteristics of the fracture and the functional outcome.

## Materials and methods

Patients being at least 18 years of age and receiving surgical treatment of an isolated TPF subsequent to a standard two-level radiograph (anterior–posterior and lateral view) and a computed tomography between January 2012 and December 2016 were identified by chart review and invited to participate in this retrospective evaluation of the outcome. The study was approved by the local ethics committee and all participants gave written informed consent prior to enrollment. Patients with malignant disease, concomitant pathological fractures, and/or impaired cognitive abilities either at time of TPF or at the time of re-examination were excluded. Patients received the German version of the validated and standardized “Knee and Osteoarthritis Outcome Score” (KOOS [9, 10]) together with an invitation to present for an assessment of the clinical and functional outcome.

### Study investigations

Demographic characteristics at time of TPF and time of follow-up examination, the 2 classifications of the fracture (AO/OTA and Luo), and the surgical approach were gathered by chart review and interview, respectively. At the time of re-examination, both legs and knee joints were investigated for asymmetry of the leg axes, morphology of the periarticular soft tissues, presence of edema, Zohlen sign, stability of ligaments, and menisci. A goniometer was used to quantify the range of motion for both knees.

### Patient-reported measure of outcome

The KOOS contains 42 questions in five subcategories (symptoms *n* = 7, pain *n* = 9, activities of daily living *n* = 17, sports activities/leisure time *n* = 5 and quality of life *n* = 4). For each item, a Likert scale is used to indicate the severity of symptoms from absent (4 points) to worse (zero points) and per subcategory, the final results are expressed on a scale from 100 (best outcome, no limitations at all) to 0 (worst case). Per subcategory and participant, results were only taken into account when at least half of the questions were addressed.

### Assessment of mobility and walking abilities

Patients performed the timed “Up & Go test” (TUG) essentially as described [11, 12]. Results were ranked as normal (≤ 10 s), mild (< 20 s), moderate (< 30 s), or severe (≥ 30 s) impairment of mobility.

Walking abilities were investigated in a standardized manner and under conditions of daily life by means of the loadsol^®^ system (novel, Munich, Germany). We decided for insoles fitted with one capacitive sensor covering the entire plantar surface of the foot. Normal plantar force during standing and walking was recorded (sampling rate 80 Hz) via miniature electronics. Data were wirelessly and in real time transferred via Bluetooth^®^ to a mobile device. The concomitant software (loadsol^®^-app) recorded cadence, loading rate, peak force, and contact time together with the force–time integral. The type of shoes was identical for all patients (sneakers, Baur GmbH Burgkunstadt, Germany) and each participant was fitted with an adequately sized and instrumented pair of shoes. After familiarization, data were recorded during in-house level walking (80 m), stair climbing (12 stairs down and up), and outdoor level walking (walkway, cobblestone, natural soil; total length of 250 m). Walking aids were allowed throughout this examination and patients walked at self-selected speed. Data were recorded for at least one minute during level walking and 0.3 min during stair climbing. To investigate symmetry of gait, we compared the step averaged contact time as well as the averaged normal ground reactions forces on the plantar surface between the unaffected and previously fractured leg. Both of these parameters are summarized in the force–time integral (FTI) which was used to calculate the factor of imbalance (FOIB; Eq. 1) as a measure of gait symmetry and functional outcome with “0” and “1” representing perfect symmetry and asymmetry, respectively.

1 $$FOIB = \frac{abs\left({FTI}_{L}-{FTI}_{R}\right)}{{FTI}_{L} + {FTI}_{R} }$$

### Data analysis and statistics

The SPSS statistical package 25.0 (SPSS Inc. Chicago, IL) was used throughout. Categorial variables are presented as frequencies, continuous variables are given as median and range. For inter- and intraindividual comparisons, the Mann–Whitney *U* test and Wilcoxon signed rank-test were used. The non-parametric Spearman rank correlation coefficients were calculated to investigate associations between the measures of outcome and relative to the interval between fracture and follow-up examination. All tests were performed two sided and a *p* value < 0.05 was considered significant.

## Results

Out of 132 patients deemed eligible by chart review, 105 were successfully contacted and invited to provide data for this retrospective analysis. However, only 58 (27 males) of these patients returned the KOOS questionnaire and consented to participate in the clinical examination. Patients presented with a median age of 58.5 [26.0–86.0] years and a BMI of 25.9 [17.1–37.8] kg/m^2^, respectively. Tibial fractures were due to high- and low-energy accidents in 42 and 16 cases. Patients with a high-energy accident were slightly younger compared to those with a low-energy trauma (52.5 [23–80] years vs. 59.0 [35–75] years; *p* = 0.043). The median time to follow-up was 3.05 [0.96–6.11] years and 2.56 [1.00–6.041] years in patients with a high- and low-energy accident, respectively. The classification of the fractures according to AO/OTA and Luo is presented in Table 1. In about half of the patients, either a single or a combined surgical and/or arthroscopically guided approach was used, while a solely arthroscopically guided treatment of the fracture was possible in only 2 patients. Regardless of the surgical approach, ligament refixation was performed as appropriate. During the post-operative period, complications (compartment syndrome (*n* = 5), prolonged healing (*n* = 5), bleeding (*n* = 4), superficial infections (*n* = 3), and thrombosis (*n* = 1)) were observed in 11 patients and in 8 of those TPF was due to a high-energy accident. Regardless of this, all complications were successfully treated in all of them. 8 and 3 patients experiencing high-and low -energy accidents, respectively.

**Table 1:** Frequencies of the fractures according to AO/OTA and Luo and the cause of the injury

	High-energy trauma	low-energy trauma
AO/OTA classification		
A1 (A1.1, A1.2, A1.3)	0/0/1	0/0/1
B1 (B1.1, B1.2, B1.3)	4/0/0	1/0/2
B2 (B2.1, B2.2, B2.3)	0/2/0	0/2/0
B3 (B31, B3.2, B3.3)	12/2/6	6/0/0
C1 (C1.1, C1.2, C1.3)	1/1/2	0/1/0
C2 (C21, C22, C2.3)	0/1/0	0/0/0
C3 (C31, C32, C33)	8/1/1	2/0/1
Luo classification		
Zero column	3	3
One column		
Lateral/medial/dorsal	9/0/5	6/0/0
Two columns		
Lateral + medial	1	0
Lateral + dorsal	11	3
Medial + dorsal	2	2
Three columns	11	2

Five patients (3 males) aged 63, 77, 65, 86, and 75 years of age and presenting for the study examinations 2–6 years after fracture used crutches (*n* = 2) or wheeled walkers (*n* = 3). Two patients out of this 5 experienced a low-energy fracture.

### KOOS results

**Fig. 1:**
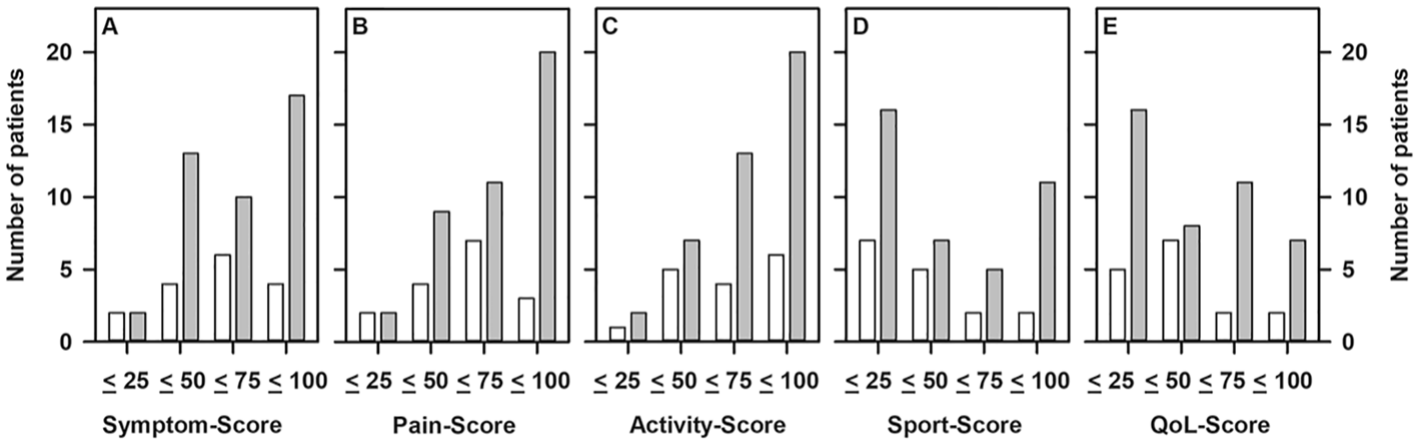
Distribution of KOOS results for the subcategories symptoms (**A**), pain (**B**), activity (**C**), sport (**D**), and quality of life (**E**). The frequency of scores per quartile is presented with “100” representing the best and “0” representing a worst outcome, respectively. Open bars: data from patients with a low-energy injury (*n* = 16); filled bars: data from patients with a high-energy injury (**A**–**C** and **E**: *n* = 42; D: *n* = 39)

All patients answered the questions referring to the subcategories “symptoms”, “pain”, “activities of daily live”, and “quality of life”. Data on sports was not provided by 3 patients (1 male) aged 61, 63 and 86 years and being examined 4 to 6 years after fracture. All of them experienced a high-energy trauma and two of them used walking aids. Results of the KOOS are summarized in Fig. 1. While the majority of our patients was fairly satisfied with the outcome in terms of “symptoms”, “pain” and “activities”, this was not the case in the dimensions “sport” and “quality of live”. Scoring of the outcome was neither related to the time of follow-up nor to the type of injury, i.e., a high- or low-energy trauma.

### Clinical assessment and evaluation of mobility

**Fig. 2:**
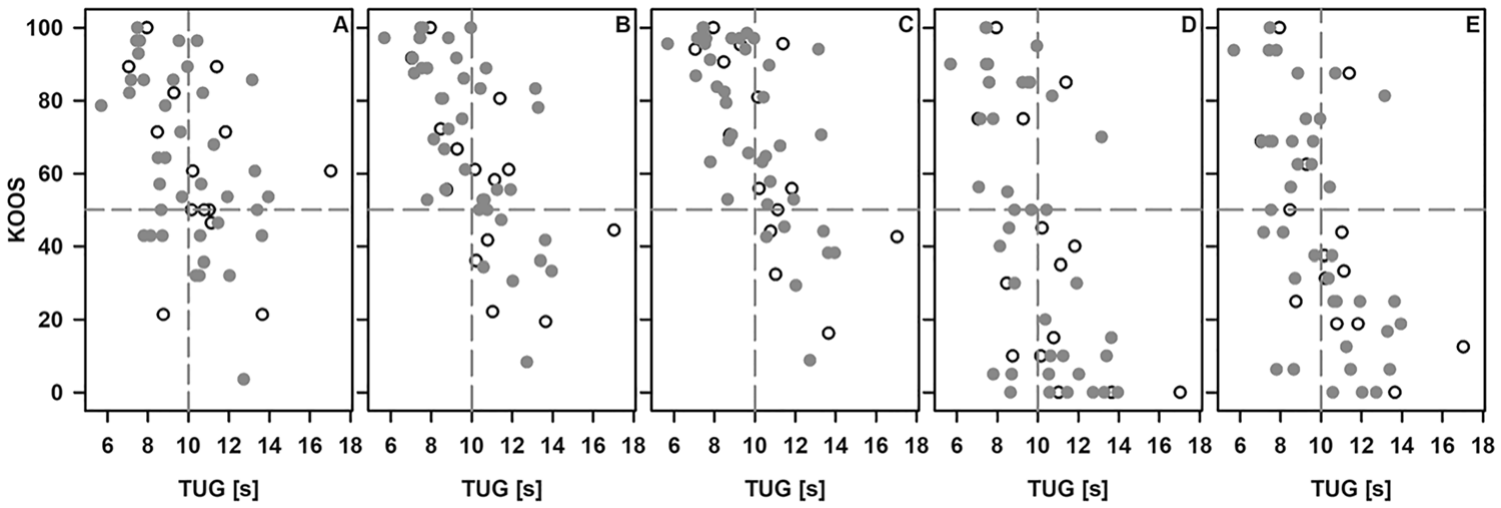
Rating of the outcome in the perception of the patient (KOOS) regarding the subcategories symptoms (**A**), pain (**B**), activity (**C**), sport (**D**), and quality of life (**E**), and mobility in terms of the TUG. Data from patients depending on walking aids are excluded. The vertical line indicates the upper limit for diagnosis of a preserved mobility and the horizontal line represent a score of 50. Open circle: data from patients with a low-energy injury (*n* = 14), filled circle: data from patients with a high-energy injury (*n* = 55)

The findings from the clinical examination of the knee are summarized in Table 2. The TUG revealed severely (48.7 s) and moderately (23.0 s) impaired mobility in two patients (two males, 58 and 62 years of age at time of TPF, five and three years after three-column TPF; AO/OTA C3.1 and C1.3) and both experienced a high-energy accident. Mobility was mildly impaired (11–19 s) and without any impairments (≤ 10 s) in 29 and 27 patients, respectively. Categorization of the patients according to preserved (TUG ≤ 10 s; *n* = 27) and impaired (TUG > 10 s; *n* = 31) mobility revealed C-type fractures in 6 and 13 patients, respectively. Considering the classification of Luo, we noted 4 and 9 three-column fractures in both groups. Results of the TUG were neither related to the type of fracture, the cause of the fracture, nor the time to follow-up. However, we noted a fairly strong association between the TUG and the patient reported outcome (Fig. 2, Supplemental Table 1).

**Table 2:** Results from the clinical examination of the knee (frequency) and TUG (median, range) in all patients

Clinical tests	Frequency of findings
Leg axes	
Anatomic/varus/valgus	36/7/15
Limb contours	
Without pathology/thickened/swollen	44/2/12
Knee joint effusion	
Negative	51
Positive at site of ptfx /non-ptfx/both	5/1/1
Zohlen sign	
Negative	50
Positive at site of ptfx/non-ptfx/both	2/5/1
Stability of the anterior cruciate ligament^*^	
Preserved	39
Impaired at site of ptfx/non-ptfx/both knees	13/0/5
Stability of lateral ligaments	
Preserved	48
Impaired at site of ptfx/non-ptfx/both knees	10/0/0
Meniscus signs^#^	
Negative	51
Positive at site of ptfx / non-ptfx/both	4/1/0
TUG	
No walking aids (*n* = 53)	10.0 s [5.69–17.0]
Wheeled walkers (*n* = 3)	14.5 s [14.2–23.1]
Crutches (*n* = 2)	17.4 s/48.7 s

*, # Due to pain these examinations were not possible in one and two patients, respectively

### Walking abilities

While all patients were capable of level walking in-house, three patients out of five patients using crutches or wheeled walkers were unable to climb stairs and refused outdoor walking with the loadsol^®^-system. For each participant and condition, we averaged the mean contact time and mean ground reaction force per leg from 90 ± 14, 26 ± 5, and 46 ± 5 steps, respectively.

**Fig. 3:**
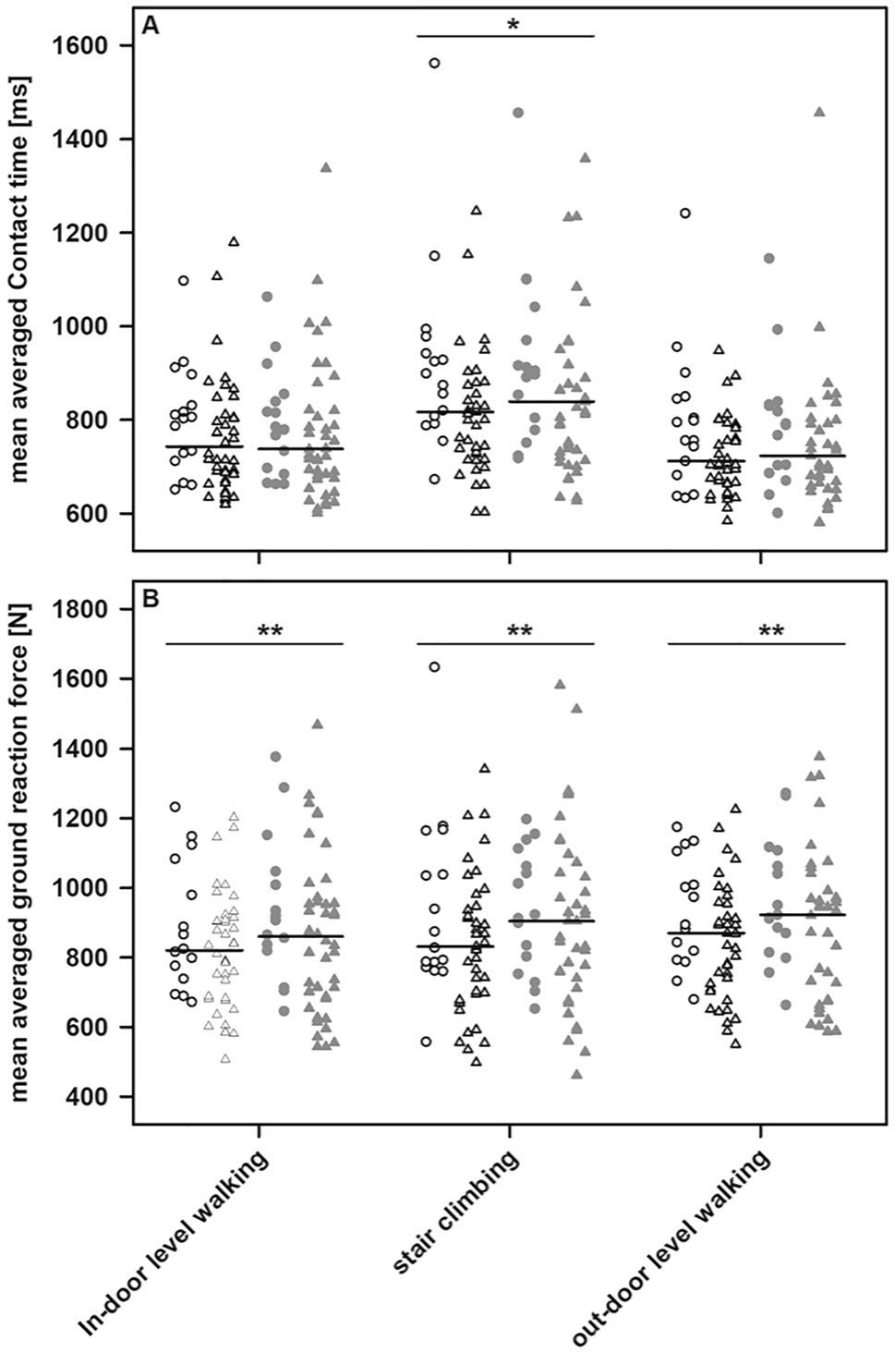
The mean averaged contact time per step (**A**) and the mean averaged ground reaction forces (**B**) for the previously fractured (open symbols) and unaffected leg (filled symbols) during in-house level walking, stair climbing, and outdoor level walking. Open circles and triangles represent data obtained from the leg experiencing a low- and high-energy trauma, respectively. Filled symbols refer to data from the unaffected leg. Median averaged contact time and ground reaction forces for the affected and unaffected legs as well as significant differences are indicated (*p < 0.05; **p < 0.005)

**Fig. 4:**
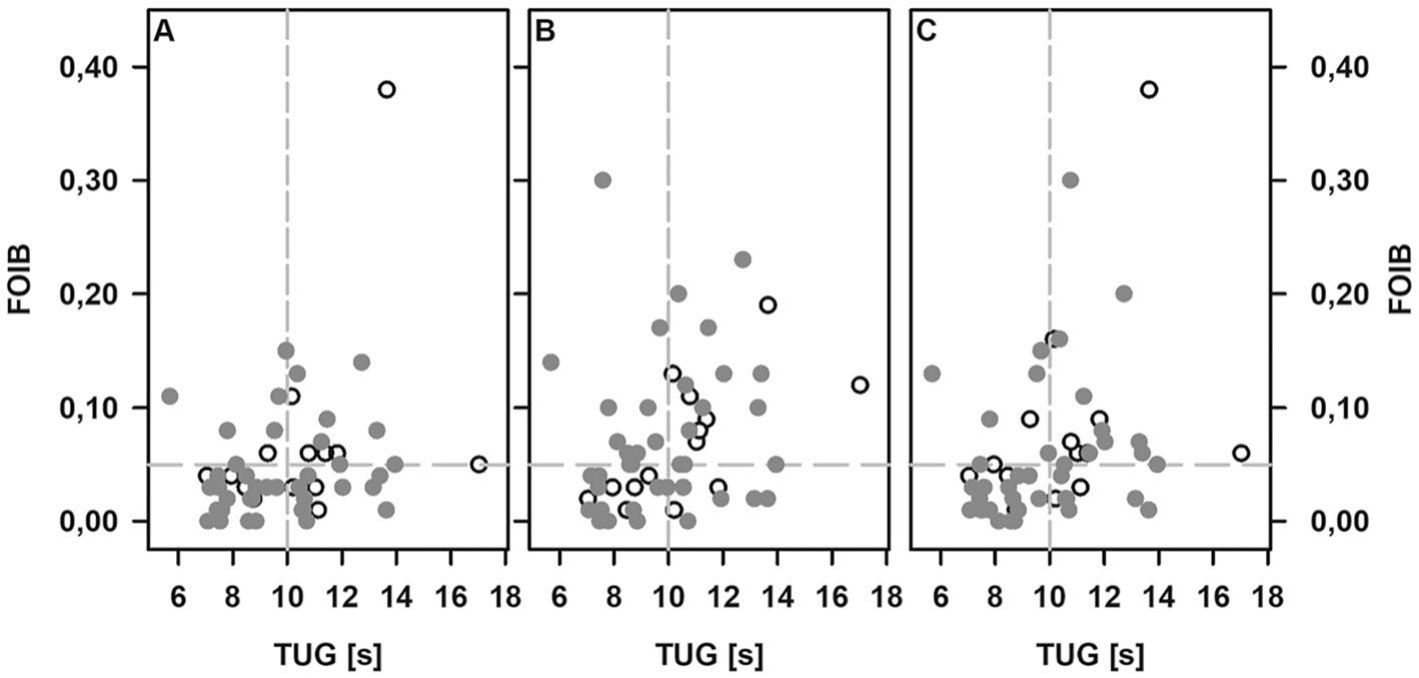
Scatter plot for visualization of the FOIB during indoor level walking (**A**), stair climbing (**B**), and outdoor level walking (**C**) relative to the results of the TUG. Data from patients depending on walking aids are excluded. The vertical line indicates the upper limit for diagnosis of a preserved mobility and the horizontal line represent a FOIB of 0.05. Open circle: data from patients with a low-energy injury (*n* = 14), filled circle: data from patients with a high-energy injury (*n* = 55)

Although limping was hardly visible even in patients using walking aids, discrepancies between the median step averaged ground reaction forces and contact times per leg were noted. In particular, the step averaged contact time during stair climbing as well as the averaged ground reaction forces recorded for the previously fractured leg were significantly lower than for the unaffected one under all conditions (Fig. 3). Next, we considered the FOIB as an individual measure of the walking abilities and evaluated the putative relation to both, the patient’s perception of outcome and the TUG. While the FOIB recorded during indoor level walking was not related to the KOOS, a moderate but significant inverse correlation with either dimension of the KOOS (Spearman rank correlation coefficients of − 0.40 (symptoms, pain, activities), − 0.39 (sport), and − 0.46 (quality of life); each *p* ≤ 0.005) and the FOIB during stair climbing was detected. Except for the category “symptoms”, this holds true for the FOIB recorded during outdoor level walking, as well (Spearman rank correlation coefficients of − 0.38 (pain, quality of life; each *p* < 0.005), − 0.33 (activities; *p* < 0.05), and − 0.29 (sport; *p* < 0.05)). Leaving out the data from patients depending on walking aids and thus looking at patients with preserved or mildly impaired mobility only, revealed a good correlation between TUG and the FOIB during outdoor level walking, while only moderate correlations of the TUG with the FOIB recorded either during in-house level walking or during stair climbing were noted (supplemental Table 1, Fig. 4).

The FOIB was neither related to the type of fracture nor to the time to follow-up. However, the FOIB during outdoor level walking tended to be higher in patients with a low-energy injury compared to those with a high-energy injury (0.06 [0.01–0.38] vs 0.04 [0.00–0.30]; *p* = 0.05).

## Discussion

We analyzed the outcome after surgical treatment of an isolated TPF in terms of the KOOS and walking abilities including the TUG. While we retrieved all patients with information about the fracture classification and chosen intraoperative approaches in order to provide data for a retrospective multicenter analysis on the association between fracture classification and surgical approach, we took the opportunity to invite our patients for an investigation of the outcome. Roughly half of the patients responding to our invitation were interested in both, reporting on the outcome to the clinicians and receiving feedback on the outcome from a clinical point of view. The vast majority of these patients experienced a TPF secondary to a high-energy injury and this holds especially true for those depending on walking aids. Although categorization according to the type of fracture or the surgical approach revealed no differences between patients with high- and low-energy fracture. Within our patient cohort, post-operative complications were predominantly seen in patients experiencing a high-energy injury.

The KOOS data revealed that the majority of patients ranked the outcome in terms of “symptoms”, “pain” and “activities” quite high while the opposite happened for the categories “sport” and “quality of life”. Although it appears reasonable to expect that the perception of the outcome differs between patients experiencing a high- and low-energy injury, our data do not support this point of view. On the one hand, the instructions for filling in the questionnaire are fairly detailed. On the other hand, the examiner is more or less blind with respect to the situation of the patient, e.g., his mood and activities before sitting down and filling in the form. In this regard, the data obtained from the TUG and loadsol^®^ assessment might help to identify those, in which a rather low KOOS, especially in terms of “quality of life”, might be independent from the overall mobility. The TUG is a simple measure for overall mobility and allows for an objective evaluation of the patient and, thus, might help to adjust the perception of the patient on the individual performance.

There are few studies reporting characteristics of gait, i.e., spatiotemporal parameters, ground reaction forces, kinematics, and/or kinetics in patients with a previous TPF [13–19]. These studies differ with regard to the design (prospective, retrospective, single or multiple assessments) and the number of patients investigated ranged from 9 to 25. Although the incidence of TPF is quite low, our own experience confirmed that follow-up of these patients is not easy to achieve as only a minority is willing to spend time for additional assessments and/or filling a questionnaire. In this regard, convincing patients to participate in a 3D gait analysis is challenging in several aspects. First of all, the equipment as well as recording and interpretation of data is not self-explanatory. Secondly, performing gait analysis is time-consuming for both, the investigator and the patient, as the latter has to be “labeled” with special markers for motion tracking of joints and limbs. Finally, instrumented gait analysis usually takes place in an artificial setting, i.e., level walking in a laboratory environment. Although 3D gait analysis requires reasonable efforts, results of all of these studies point to asymmetry in gait. In view of the aforementioned obstacles related to 3D gait analysis we decided for an easier approach, i.e., the utilization of insoles and assessment of walking abilities under conditions of daily life. This approach revealed that asymmetry of gait is most obvious during stair climbing and outdoor level walking. Thus, it might be an option to consider not only the TUG but also simple analysis of walking abilities by means of insoles to objectify the patient’s perception of poor outcome. In particular, climbing stairs up and down and outdoor walking using insoles for assessment reveals any functional deficits after TPF much better than analysis of indoor walking in a conventional gait lab and provides a profound insight into the daily problems experienced by patients after TPF.

Our study certainly has some limitations. First, due to the retrospective study design; second, due to the bias in patient selection and a disparity in age distribution between the groups without and those with a moderate gait disturbance, respectively; third, due to the assumption that only those with high interest in their own health responded to the invitation for assessment. The strengths of the study are based on the high number of individuals recruited for functional evaluation and clinical assessment and the bundle of assessment tools employed.

## Conclusion

Descriptive parameters as the two fracture classifications of the AO/OTA and Luo are not relevant for predicting functionality following TPF. The same holds true for the interval from fracture treatment to the time-point of examination. A simple clinical test as the TUG is able to mirror functional deficits quite well. Simple gait analysis techniques relying on the analysis of different tasks appear to yield a more sophisticated image on functional deficits after TPF than classical exam of ground-level walking and correlate quite well with validated patient-related outcome measures as the KOOS.

### Supplementary Information

Below is the link to the electronic supplementary material.

Supplementary file1 (DOCX 12 KB)

## Data Availability

The dataset supporting the conclusions of this article is available from the corresponding author on reasonable request.
