# Supplementary material for: Functional assessment and outcome following surgical treatment of displaced tibial plateau fractures: a retrospective analysis
Source: Eur J Trauma Emerg Surg. 2023 Nov 17;49(6):2373–9. doi: 10.1007/s00068-023-02401-x (PMC10728243; doi:10.1007/s00068-023-02401-x)
Supplement: Supplementary file 1 — Supplementary file1 (DOCX 12 KB) [file 68_2023_2401_MOESM1_ESM.docx]

**Supplemental Table 1: Pairwise Spearman rank correlation coefficients between results from the TUG with either the KOOS or the FOIB**

|  |  | TUG |
| --- | --- | --- |
| KOOS | |  |
|  | Symptoms | -0.48; p<0.001 |
|  | Pain | -0.70; p<0.001 |
|  | Activities | -0.71; p<0.001 |
|  | Sport | -0.61; p<0.001 |
|  | Quality of Life | -0.60; p<0.001 |
| Loadsol® data | |  |
|  | FOIB_indoor level walking_ | 0.33; p<0.05 |
|  | FOIB_stairs_ | 0.32; p<0.05 |
|  | FOIB_outdoor level walking_ | 0.44; p<0.01 |

Data from patients with walking aids are excluded.
